# Efficacy and Safety of Setmelanotide, a Melanocortin-4 Receptor Agonist, for Obese Patients: A Systematic Review and Meta-Analysis

**DOI:** 10.3390/jpm13101460

**Published:** 2023-10-04

**Authors:** Bárbara Ferraz Barbosa, Francisco Cezar Aquino de Moraes, Camila Bordignon Barbosa, Plínio Takashi Karubi Palavicini Santos, Izael Pereira da Silva, Bruno Araujo Alves da Silva, Jamile Cristine Marques Barros, Rommel Mario Rodríguez Burbano, Ney Pereira Carneiro dos Santos, Marianne Rodrigues Fernandes

**Affiliations:** 1 Department of Medicine, University of Aquino Bolivia, Santa Cruz de la Sierra 0701, Bolivia; bferraz.barbosa@hotmail.com (B.F.B.); bordignoncamila4@gmail.com (C.B.B.); 2 Oncology Research Center, Federal University of Pará, Belém 66073-005, PA, Brazil; jamilecbarros@gmail.com (J.C.M.B.); npcsantos.ufpa@gmail.com (N.P.C.d.S.); fernandesmr@yahoo.com.br (M.R.F.); 3 Department of Medicine, Faculty of Medicine of Bauru, University of São Paulo, Bauru 17012-230, SP, Brazil; pliniopalavicini@usp.br; 4 Department of Medicine, Federal University of Amazonas, Manaus 69020-160, AM, Brazil; izaelbatista999@gmail.com; 5 Department of Medicine, State University of Ceará, Fortaleza 60714-903, CE, Brazil; bru.araujo@aluno.uece.br; 6 Department of Medicine, Otávio Lobo Children’s Cancer Hospital, Belém 66063-240, PA, Brazil; rommelburbano@gmail.com

**Keywords:** setmelanotide, obesity, MC4R agonist, RM-493

## Abstract

Background: A malfunction in the melanocortin-4 receptor (MC4R) is associated with obesity in rare genetic syndromes; setmelanotide is a new drug that activates this receptor and is being used to treat severe obesity. This meta-analysis evaluated the efficacy and safety of setmelanotide for weight loss in severe obesity linked to human MC4R deficiency. Methods: We searched PubMed, Embase, and Cochrane for randomized and nonrandomized clinical trials using setmelanotide. We considered a *p*-value ≤ 0.05 statistically significant. Results: We included 376 patients, of whom 328 (87.2%) received setmelanotide for a mean follow-up of 52 weeks. The mean age was 32.8 (14.67) years. Weight loss was significant (MD −3.52; 95% CI −3.98, −3.05; *p* = 0.01; I^2^ = 92%), with an average proportion of −6.91% weight loss during treatment. Changes in BMI showed an MD of −10.55 kg/m^2^ in patients > 18 years and −0.61 kg/m^2^ in patients < 18 years (BMI score). However, the drug was associated with a higher risk of skin hyperpigmentation (OR 0.69; 95% CI 0.55, 0.80; *p* = 0.08). Conclusions: Our results support the use of setmelanotide in treating severe obesity.

## 1. Introduction

The World Health Organization (WHO) considers obesity to be a serious global public health problem. By 2025, 2.3 billion adults are expected to be overweight and 167 million people will suffer the consequences of obesity [1,2]. According to the Obesity Atlas 2023, by 2035, more than 4 billion people could be affected, reflecting an increase from 38% in 2020 to 50% in 2035, with a prevalence of obesity of 24% of the population, more pronounced among children and adolescents [3].

Obesity, a chronic disease that represents an imbalance between energy intake and expenditure, is difficult to control due to the high rates of failure in the therapeutic approach linked to diet and physical activity [4,5]. Therefore, weight control drugs are great allies in reducing body measurements, with appetite modulation strategies, food suppressants, and peripherally acting compositions [6].

Some genetic diseases, such as Bardet–Biedl and Alström syndromes, have their ciliary signaling altered by a component of the leptin–melanocortin pathway (melanocortin-4 receptor (MC4R)); this plays a role in regulating body weight, and its failure contributes to hyperphagia and obesity, even at an early age [7,8,9]. The estimated frequency of morbidly obese people with MC4R receptor alterations is approximately 1–4% in children and adults [10,11]. Heterozygous loss-of-function (LOF) mutations in MC4R have been observed with a higher frequency in children or adults with severe obesity. As a functional consequence, each mutation results in a complete LOF, a partial LOF, or no LOF (wild type) [12].

Recent randomized studies with setmelanotide (RM-493), a synthetic cyclic peptide that binds to human MC4R with high affinity, have demonstrated significant reductions of approximately 10% body weight [8,11,12,13]. Thus, setmelanotide is the first drug approved by the Food and Drug Administration (FDA) for the treatment of severe obesity of genetic origin [13].

Despite promising previous studies with setmelanotide, there is still no consensus on its effectiveness in reducing weight, appetite, and side effects in obese patients, with and without associated genetic syndromes. We therefore carried out a meta-analysis to assess the efficacy and safety of this therapy for the treatment of obesity.

## 2. Materials and Methods

### 2.1. Protocol and Registration

This meta-analysis followed the guidelines of the Preferred Reporting Items for Systematic Reviews and Meta-Analysis (PRISMA) statement and the recommendations of the Cochrane Collaboration (Page et al.) This review was registered with the Prospective International Registry of Systematic Reviews—PROSPERO (http://www.crd.york.ac.uk/, accessed on 2 September 2023)—under number CRD42023416805.

### 2.2. Eligibility Criteria

We included (1) randomized and non-randomized clinical trials; (2) investigating setmelanotide in patients with severe obesity; (3) studies reporting adverse effects during treatment. We excluded (1) articles such as letters, editorials, expert opinions, reports, case series, reviews, and animal model studies; (2) studies without outcomes of interest; (3) those with an overlapping population; (4) those with a follow-up time of less than 1 month; (5) those without complete published results; and (6) studies reported and published in non-English.

### 2.3. Data Extraction Search Strategy

We systematically searched PubMed, Embase, and Cochrane Central for articles published up to 23 April 2023. We also reviewed the records of the Journal of the Endocrine Society (JES). The following search terms were used: “obesity”, “obese”, “Bardet-Biedl syndrome”, “Alström syndrome”, “setmelanotide”, “MC4R”, “melanocortin 4 receptor”, “melanocortin”. The references of all included studies were also manually searched for additional studies. Two authors (F.B. and S.B.) independently carried out the literature search and extracted the data following the eligibility criteria described above. In the event of disagreements between the reviewers, a third reviewer was responsible for the final decision (F.M.).

### 2.4. Outcomes and Subgroup Analysis

The primary endpoint was the percentage change in body weight after maximum treatment at the therapeutic dose in participants completing the treatment phase. Secondary endpoints included body mass index (BMI) (>18 years), BMI score (<18 years), hunger score, waist circumference-related variables, safety, and tolerability capacity (as assessed by the frequency and severity of adverse effects (AEs) changes in laboratory tests (lipids), vital signs (blood pressure), and injection site reactions (ISRs)) over the course of treatment.

### 2.5. Quality Assessment

Most of the included studies were single-arm or uncontrolled studies, so the tools of the Newcastle–Ottawa Scale (NOS) [14] were used to assess the quality of the included studies [7,15,16,17,18,19,20,21,22]. In this scale, studies are scored on a scale from 0 to 9, according to the quality of the selection, the comparability of the groups, and the attribution of the results. The quality of the randomized clinical trials was assessed using the Cochrane Collaboration’s tool [23] for the assessment of the risk of bias in randomized trials, in which studies are classified according to risk as high, low, or unclear in 5 domains: selection bias, performance, detection, attrition, and reporting. The Grading of Recommendations, Assessment, Development, and Evaluation (GRADE) [24] tool was used to rate the certainty of the evidence in this review as high, moderate, low, or very low. The strength of recommendations was graded using the GRADEpro Guideline Development Tool (https://gdt.gradepro.org/, accessed on 2 September 2023).

### 2.6. Statistical Analysis

Odds ratios (OR) with 95% confidence intervals were used to compare treatment effects for categorical outcomes, and continuous outcomes were compared with mean differences (MD). Cochran’s Q test and the I^2^ statistic were used to assess heterogeneity; *p* values of less than 0.10 and I^2^ > 25% were considered significant for heterogeneity. We used a fixed-effect model for results with low heterogeneity (I^2^ < 25%). Otherwise, a DerSimonian and Laird random-effects model was used. We also carried out a sensitivity analysis with meta-regression. Review Manager 5.3 (Cochrane Centre, The Cochrane Collaboration, Denmark) and Open Meta-Analyst (CEBM @ Brown) [25] were used for the statistical analysis.

## 3. Results

### 3.1. Study Selection and Baseline Characteristics

**Figure 1:**
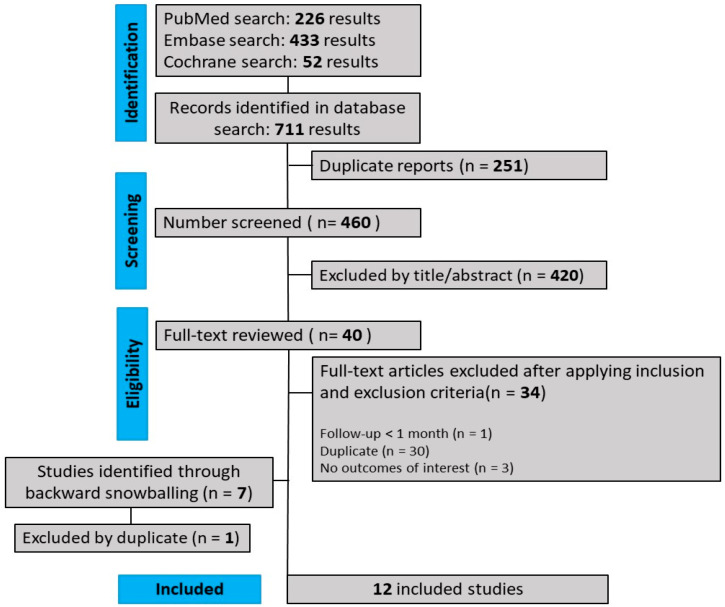
PRISMA flow diagram of study screening and selection.

We found 711 titles, which, after removing duplicates and ineligible studies, left 47, which were analyzed based on the eligibility criteria described above. Of these, a total of 12 studies were included, comprising 376 patients (Figure 1).

The mean age was 32.8 (14.67) years [17,19,21,22,26], with a prevalence of females (72%) [7,8,21,22,26]. A total of 328 (87.2%) patients received setmelanotide. The characteristics of the studies are shown in Table 1.

Anthropometric, cardiovascular, and metabolic data (Table S1) were calculated comparing the baseline parameters with the reduction shown at approximately 1 year of treatment with setmelanotide, showing positive results, with a decrease in hunger scores of −28.0% (30.8) and mean abdominal wall circumference of −6.31% (8.50); in laboratory parameters, positive clinical changes were observed in the lipidogram and maintenance of blood pressure values, which reaffirmed the safety of the drug.

### 3.2. Pooled Analyses of All Studies

The statistical analysis of the efficacy and safety of setmelanotide for the treatment of severe obesity is summarized in Table 2. The outcome with the highest number of events in the patients included was weight loss (197), followed by hyperpigmentation (131), which proved to be the adverse effect of the greatest clinical importance.

#### 3.2.1. Effectiveness

##### Weight Loss

**Figure 2:**
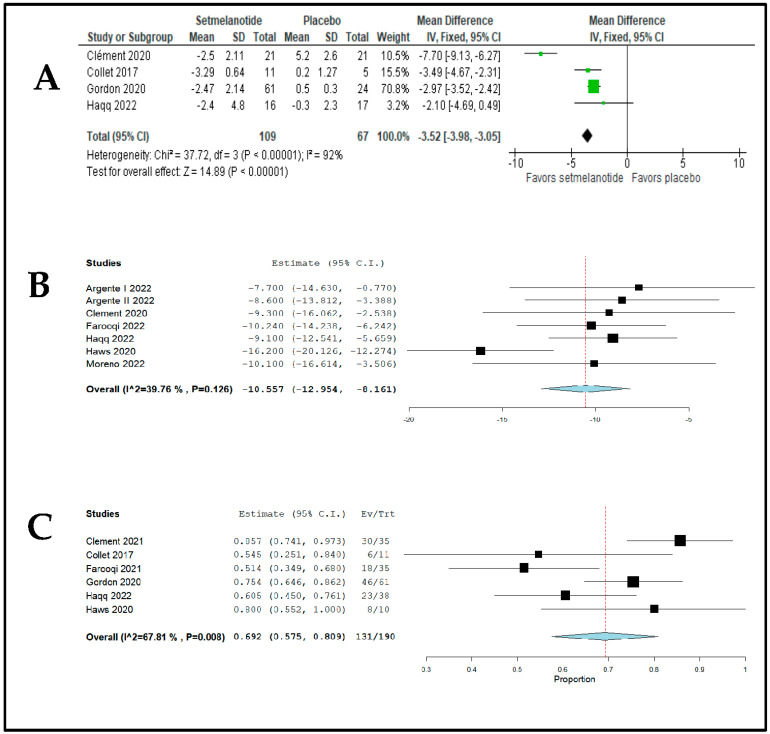
Forest plots showed that weight loss was significant in the setmelanotide group (**A**) [7,8,12,26], with a combined mean difference in BMI: ORR −10.55 kg/m^2^ (95% CI: −12.95, −8.16; I^2^ = 39.76%) (**B**) [7,8,15,16,18,20,22] and risk of skin hyperpigmentation (ORR 0.69; 95% CI 0.55, 0.80; *p* = 0.08) (**C**) [8,12,17,19,22,26].

Four studies with 176 patients reported the mean difference in weight loss (setmelanotide versus placebo), analyzing the population over a period of 4 to 14 weeks. The pooled mean was −3.52% (95% CI −3.98%, −3.05%; *p* < 0.00001), with significant heterogeneity (I^2^ = 92%; *p* < 0.00001) (Figure 2A).

##### Average Weight and BMI Reduction

Eleven studies [7,8,12,15,16,18,19,20,21,22,26] evaluated the efficacy of treatment with setmelanotide for weight loss; seven studies [7,8,16,17,18,20,22] analyzed the population over a total of 52 weeks. Collet (2017) [12] evaluated them over 4 weeks, and Farroqi (2021) [19] and Gordon (2020) [26] over 12 weeks. In the Clément (2018) [21] study, the authors used an average of 35 weeks. The dose of medication used varied from 0.01 mg/kg/day [12] to 30 mg/week [26], settling at an average of 2.5–3 mg/day in most studies [7,8,17,19,21,22,26].

The mean difference in percentage (% mean) weight loss for the pooled data was −6.91% (95% confidence interval (CI); −8.48, −5.34; I^2^ = 92.04%; Analysis 2; Figure S1) and *p*-value < 0.001. Seven studies [7,8,15,16,18,20,22] reported the BMI (>18 years) and BMI score (<18 years) to assess the efficacy of treatment in reducing body mass index in kg/m^2^, while the Haws study [22] did not study patients under the age of 18, and did not contribute statistically to the meta-analysis regarding the BMI score. The combined mean difference in BMI score was a decrease of −10.55 kg/m^2^ in body mass index in obese patients over the age of 18 (95% CI: −12.95, −8.16; I^2^ = 39.76%, Analysis 3; Figure 2B).

In the population of patients under 18 years of age, the data showed an average reduction of −0.61 kg/m^2^, with a *p*-value < 0.001 and heterogeneity of 99% (Analysis 4; Figure S2).

##### Hunger Score

The mean percentage difference (% mean) in the decrease in the maximum hunger score for the grouped data was −35.6% (95% confidence interval (CI) −60.61%, −10.61%; I^2^ = 97.73%; Analysis 5; Figure S3).

#### 3.2.2. Safety

Hyperpigmentation was the most common adverse effect, with 131 (68.9%) patients affected. An injection site reaction occurred in 78 (41.05%) and was also reported in the studies that used a comparison with a placebo, the specific use of the medication not being a risk factor (Table S2).

One treatment-related serious adverse event occurred in a patient receiving a placebo in the study [8]; five serious adverse events (depression, major depression, adrenocortical insufficiency, pneumonia, and pleurisy) were reported in four study participants [7], but their relationship with setmelanotide was not considered. Four serious adverse events (cholecystitis, suicidal ideation, and gastric band reversal) were reported in three participants, also unrelated to treatment with setmelanotide. One participant dropped out of the study because of hypereosinophilia, which was considered possibly related to setmelanotide treatment and resolved after discontinuation. In the case of suicidal ideation, Clément [7] indicated that the treatment may have had a factor linked to an increase in pre-existing depression. Setmelanotide was generally well tolerated. All participants reported at least one drug-related AE in the Haws study [22].

In total, 177 adverse effects were reported in 179 patients; 14 patients left the studies (total denominator of 268 patients) as a result of side effects that were not considered to be related to treatment with setmelanotide.

The meta-analysis assessing the risk of skin hyperpigmentation was feasible with the inclusion of 6 [8,12,17,19,22,26] of the 12 studies, including a total of 190 patients who suffered 131 events. The Clément (2020) [7] study was not included due to overlapping patients in this outcome; the Argente I [15], Argente II [16], Farooqi [18], and Moreno [20] studies did not present data for the outcome of interest. The risk ratio for this adverse effect was 69% (95% confidence interval: 0.57, 0.80; Analysis 6; Figure 2C), showing statistical significance in terms of the increased risk of developing skin hyperpigmentation with the use of setmelanotide (*p* < 0.001).

A total of five studies [12,15,17,22,26] including 155 patients presented the clinical outcome of interest. The incidence of headache may be an expected adverse effect caused by the use of the drug (*p* < 0.001), having statistical significance with a risk ratio of 27% (95% CI: 0.12, 0.43, I^2^ = 81.38%; Analysis 7; Figure S4), occurring in 50 included patients. Of the included studies, six [8,12,17,19,22,26] reported the number of occurrences of nausea as an adverse effect, ranging from 1 to 31 patients out of 190 analyzed. There was significant heterogeneity (I^2^ = 77%, *p* < 0.01). The analysis showed a risk ratio of 39% (95% CI: 0.24%, 0.53%; Analysis 8; Figure S5). Complications such as vomiting occurred in 38 of the 155 patients who used setmelanotide for the treatment of severe obesity. The incidence of the complication was 20% (95% confidence interval: 0.10, 0.31) for all studies. There was no statistically significant difference in the total risk of the adverse effect occurring (*p* = 0.029). There was moderate heterogeneity (I^2^ = 62.96%; Analysis 9; Figure S6). To investigate the safety of the drug in the occurrence of sexual dysfunction, an arm meta-analysis was carried out with four studies (144 patients). The heterogeneity test showed that I^2^ = 43.68% and *p* = 0.149. The statistical meta-analysis showed that the overall clinical risk rate was 7% (95% CI: 0.014, 0.135; Analysis 10; Figure S7). To investigate the safety of the drug in the occurrence of ISRs, an arm meta-analysis was carried out with three studies (106 patients) [17,22,26]; the Clément (2020) [7] study was excluded because it had the same study population as Clément (2021) [17] in this outcome. The heterogeneity test showed that I^2^ = 88.71% and *p* < 0.001. The statistical meta-analysis showed that the overall clinical risk rate was 81% (95% CI: 0.61, 1.01; Analysis 11; Figure S8).

### 3.3. Quality Assessment

The results of the assessment are shown in Table S1 and were categorized as low risk (NOS score ≥ 7 points). For randomized studies, the Cochrane Collaboration’s RoB 2 tool was used [23]; three RCTs [8,12,26] were analyzed with this tool, resulting in a low risk of bias (Figure S9).

The overall certainty of the evidence for the outcome assessed was rated as high according to the GRADE tool [24]. Figure S10 summarizes the GRADE assessment and results of this review, providing an overview of the level of certainty of the evidence for the weight loss outcome that was assessed.

### 3.4. Sensitivity Analysis

Weight heterogeneity was the greatest found, falling by 3.49% from baseline after the withdrawal of the Haws study [22] (I^2^ = 88.55%) (OR −6.68; 95% CI −8.27, −5.09; Figure S11); in the comparative meta-analysis with a placebo, the withdrawal of the Clément [7,21] and Gordon [26] studies ensured a fall of I^2^ = 0%. In the BMI analysis (I^2^ = 39.7%), there was a drop to 0% without the Haws [22] study (OR −10.55; 95% CI −12.95, −8.16; Figure S12). The BMI score (I^2^ = 99.16%) fell slightly without the Farooqi [18] study (66.11%) (OR −0.61; 95% CI −1.37, 0.15; Figure S13). In the maximum hunger score, heterogeneity dropped from 97.73% to 85% with the exclusion of Farooqi [19].

In the safety analysis, the meta-analysis of ISRs caused by the administration of setmelanotide obtained high heterogeneity, with I^2^ = 88, suffering a sharp drop (I^2^ = 0%) after the withdrawal of the Gordon [26] study (OR 0.81; 95% CI 0.61, 1.01; Figure S14); in the occurrence of hyperpigmentation (I^2^ = 67.8%), heterogeneity dropped to 50.8% without the Clément [17] study (OR 0.69; 95% CI 0.57, 0.80; Figure S15).

## 4. Discussion

Our meta-analysis showed a significant difference between the setmelanotide group versus placebo in relation to weight loss (*p* < 0.01), with a decrease in appetite (−35.6%). With regard to adverse effects, we observed that hyperpigmentation was the main associated adverse effect; this is expected in this population due to the stimulation of the melanocortin-1 receptor (MC1R), expressed in melanocytes, which plays an important role in regulating melanin synthesis in the skin [27]. The GRADE tool [24] was used to assess and categorize the certainty of evidence regarding weight loss. The result obtained showed a high level of evidence.

Several pro-obesogenic genes are expressed and/or act in the central nervous system, suggesting that neuronal components may promote or suppress energy expenditure and appetite in obesity [28]. One of these genes, expressed in the hypothalamic area and active in energy balance and appetite, is MC4R [10]. It is known that genetic inheritance is responsible for 40–75% of all cases of obesity and heredity studies provide strong evidence of a genetic contribution to obesity susceptibility [29,30]. It can be said that the genetic susceptibility to obesity is heterogeneous, whether syndromic, resulting from chromosomal rearrangements, or non-syndromic, composed of monogenic and polygenic.

Setmelanotide, an MC4R agonist, was developed for the treatment of severe obesity and appetite control [12]. These consequences are related to proopiomelanocortin (POMC), proprotein convertase subtilisin/kexin type 1 (PCSK1), or leptin receptor deficiency (LEPR), as well as other rare genetic diseases, including BBS and Alström syndrome. The drug received approval in 2020 for use in patients over the age of 6 with obesity genetically related to some MC4r deficiency; more than 200 obese patients with no known genetic association were treated with setmelanotide for a short term, with an approximate weight loss of 1 kg/week observed in up to 4 weeks, with no major adverse effects or cardiovascular changes associated with the medication [31].

Body weight reduction was observed in several animals with obesity of genetic origin treated with setmelanotide; however, the same results were not seen in MC4R knockout mice. Studies with mice have made it possible to better understand the functioning of these pathways, indicating that the effects of the drug on body weight regulation are closely linked to the types of variations and mutations in MC4R [12]. Randomized studies have shown that the drug administered by means of a daily injection promotes significant weight loss in individuals with severe obesity after one year, generating at least a 10% body weight reduction associated with improved appetite control [13]. 

Orlistat, bupropion, and liraglutide, FDA-approved drugs for weight control and obesity treatment, produce relatively low weight loss and are used in conjunction with dietary changes and a reduction in physical inactivity [32]. Until 2021, high doses of phentermine and especially topiramate were used for weight loss, with results ranging from 3.4 to 8.9 kg in 1 year (≥5% of initial weight vs. placebo) in patients with non-syndromic obesity, demonstrated for a network meta-analysis published in 2016 that compared a placebo with various weight control medications, and the results showed that phentermine-topiramate was associated with weight loss in approximately 75% of participants, as well as liraglutide (63%), naltrexone-bupropion (55%), and orlistat (44%) [33,34]. Recently, semaglutide (Wegovy), a glucagon-like peptide-1 receptor agonist, was associated with 15% body weight loss at one year in non-obese patients without associated hereditary syndrome; however, the first clinical trial (SELECT) to evaluate the cardiovascular outcomes of the drug is underway, and more information on its safety will be provided after its completion [32]. Our results showed weight loss of −3.52% (95% CI −3.98%, −3.05%; *p* < 0.00001) over a period of 4 to 14 weeks, with no known harmful cardiovascular effects.

In an additional analysis, four studies with 176 patients reported a mean difference in weight loss (setmelanotide versus placebo) of −3.52%, with statistical significance. In single-arm analyses, the combined mean difference in BMI score was a −10.55 kg/m^2^ decrease in body mass index in obese patients over the age of 18; a −35.6% decrease in hyperphagia and an improvement in laboratory parameters were also observed with the use of setmelanotide for approximately 12 months. In addition to changes in clinical and anthropometric parameters, improvements in lipid levels were noted (percentage change from baseline: HDL cholesterol 14.0852% (26.34); LDL cholesterol −8.0016% (23.65); and triglycerides −16.3839% (30.93)), as well as cardiovascular parameters (percentage change from baseline: systolic blood pressure 0.54% (10.59) and diastolic blood pressure −2.67% (13.39)), with no clinical changes or serious adverse effects in the patients studied. 

A study with only two rare patients who had POMC deficiency and were treated with setmelanotide showed satisfactory results in body weight control (patient 1: 51 kg after 42 weeks; patient 2: 20.5 kg after 12 weeks), with no significant adverse effects reported [31]. In the complete absence of POMC, the results showed that these individuals were highly responsive to treatment with setmelanotide [10,11]. 

Multicenter, open, single-arm phase 3 studies were carried out in ten hospitals in Canada, the USA, Belgium, France, Germany, the Netherlands, and the UK in patients with morbid obesity associated with pro-opiomelanocortin deficiency (POMC) or obesity due to leptin receptor deficiency (LEPR). Clément et al. [7], reported the results of these trials involving 10 patients in the POMC study and 11 in the LEPR study, with a treatment success rate equivalent to 80 per cent (POMC) and 45 per cent (LEPR), considering a body weight reduction rate of approximately 10 per cent in 1 year, with an average percentage change in the highest hunger score of −27.1 per cent (*p* = 0.0005; POMC study) and −43.7 per cent (*p* < 0.001; LEPR study), showing the drug’s efficacy in reducing body weight and hyperphagia in this population [7]. The research described by Haqq et al. [8] showed a significant weight reduction only in patients with Bardet–Biedl syndrome, being inconclusive for Alström syndrome. 

Mild adverse effects were reported by several studies; injection site reactions and increased skin pigmentation were the most common, with different percentages varying according to the population studied; nausea, headache and diarrhea were also reported to a lesser extent [8,13]. A study that separated patients with POMC and LEPR syndrome quantified the adverse reactions in this population, showing that, despite following the same line described by previous studies, hyperpigmentation was reported in 100% of individuals with POMC, followed by nausea (50%) and vomiting (30%). In the LEPR study, the reactions commonly associated with setmelanotide treatment were ISRs (100%), skin disorders (45.4%), and nausea (36.3%) [7]. Our results showed that hyperpigmentation was the most common adverse effect (68.9%), followed by nausea (44,7%) and ISRs (41.05%).

Our results showed the occurrence of gastrointestinal adverse effects (nausea (44%), vomiting (20%), and diarrhea (4%)), with a peak incidence in the first month of use; however, these same effects are expected in other drugs used for obesity control and weight loss. Similarly, treatment with Semaglutide, an analogue of glucagon-like peptide-1 (GLP-1), has gastrointestinal disorders as the main adverse effects, and, despite being mild to moderate in severity, these complaints were the main causes related to the discontinuation of treatment [35,36]. Adverse effects have also been reported in some studies with Liraglutide, including gastrointestinal disorders, those related to the gallbladder, and symptomatic hypoglycemia [37,38,39,40]. In our study, no serious adverse effects such as hypoglycemia were reported in any of the patients included.

Obesity places a physical and psychosocial burden on individuals and their families, so improving the quality of life of these families in the long term should be one of the aims of drug treatment. A phase 3 study evaluated the impact of setmelanotide treatment on BBS patients, with the majority of patients and carers describing significant clinical improvements after one year of treatment, including psychosocial and physical functioning [41]. The results of recent clinical studies are promising and show emerging compounds as a critical point where research and clinical trials could result in excellent solutions for obese patients in the future [6].

A possible interpretation of the reduction in heterogeneity between the combined results could be the inclusion of two different syndromes (BBS and Alström syndrome) in the population composition of Haqq’s study [8], for example, which differed from the population characteristics of the other studies included in the analysis. The results for Alström syndrome were not as expressive as those for Bardet–Biedl syndrome, due to the small number of carriers, which would explain the high heterogeneity of this study. It is believed that high doses of the drug in weekly administration are not related to the increased incidence of adverse effects in general; the statistical analysis of the outcome related to skin reactions caused by the administration of setmelanotide showed that, despite the doses, the Gordon study [26] represented the lowest rates of events. 

In the sensitivity analysis, the grouped data indicated that the cause of the high heterogeneity in the outcomes was closely related to the different syndromes analyzed and the treatment profile (dose/time). With regard to the high level of heterogeneity shown in most of the results, in rare disease scenarios, the randomization of studies usually guarantees homogeneous samples, which is not the case in non-randomized studies. Therefore, this is a precipitating factor to consider in addition to the others previously mentioned. However, adverse effects are mild and expected, and their occurrence does not contraindicate the use of setmelanotide.

Studies are currently being conducted with setmelanotide; the phase 3 EMANATE study (NCT05093634), with an estimated 400 patients, focuses on patients with a heterozygous variant of the POMC/PCSK1 genes, the LEPR gene, the SRC1 gene, and the SH2B1 gene, and the phase 2 DAYBREAK study (NCT04963231) aims to study approximately 10 genes in 150 individuals, being a two-stage, double-blind, placebo-controlled study over 24 months. Both are underway, and important findings may be provided upon completion [42]. 

This is the first meta-analysis to assess the efficacy and safety of setmelanotide in patients with or without genetic syndromes. The strength of this meta-analysis was the use of all available clinical studies to assess the safety and efficacy of the drug, and an additional weight analysis was carried out with placebo-controlled studies; all endpoints were included. Our results support that the addition of setmelanotide significantly increases weight loss in this population, associated with good tolerability and safety, demonstrating that its benefits outweigh its potential risks, taking into account the evidence of safety and efficacy evaluated in this study, as well as the severity of the disease that the drug is intended to treat. The size of the population sample is not a limitation, due to the feasibility of the hypothesis and the rarity of the population. Finally, the safety profile of the use of setmelanotide requires further investigation; two long-term studies are underway, and it is recommended that this meta-analysis be updated with the results of the new studies to assess the long-term effects of the drug in the near future.

## 5. Limitations

Because some studies selected for the meta-analysis had different populations, there may have been a selection bias. The excluded studies did not meet the criteria for inclusion in the meta-analysis. In addition, ongoing studies will be able to determine whether influences such as age, gender, and ethnicity impact the results. This review analyzed the efficacy and safety of setmelanotide for the treatment of obesity. The bias may have been reinforced by the fact that incomplete or erroneous data from a previous analysis were used. The values may have been accidentally distorted because of a lack of information and certain unpublished studies.

## 6. Conclusions

Our results show that setmelanotide (MC4R agonist) is associated with significant weight loss, body measurement, and hunger improvements in individuals who undergo treatment for obesity, and it is considered a safe drug with manageable adverse effects. The treatment was well tolerated in all the studies, the laboratory and clinical parameters evaluated showed considerable improvements, and the addition of setmelanotide is indicated for long-term treatment to control severe obesity and hyperphagia, especially in individuals with associated genetic syndromes.

## Figures and Tables

**Table 1:** Characteristics of the included studies.

Study (Years)	Follow-Up (Weeks)	Age, Years (Mean)	Sex *n* (%)	Syndromes	Weight, kg (Mean)	BMI, kg/m^2^ (Mean)	BMI Score kg/m^2^ (Mean)	N. of Patients (Int./Placebo)	Total	Doses (mg)	Quality Score
Argente I 2022 *^a^ [15]	78–104	NA	NA	BBS	132 (20.9)	42.2 (9.2)	3.5 (0.76)	54/-	54	NA	8
Argente II 2022 *^a^ [16]	26–52	NA	NA	SH2B1/16p11.2	139.7 (35.4)	47.2 (12.8)	3.56 (0.60)	35/-	35	NA	8
Clément 2018 [21]	45–61	19.66 (4.93)	Male: 2 (67) Fem.: 1 (33)	POMC/LEPR	124.43 (5.39)	41.6 (2.28)	NA	3/-	3	0.5–2.5	8
Clément 2020 *^b^ [7]	52	21.1 (7.74)	Male: 8 (38) Fem.: 13 (62)	POMC/LEPR	126.3 (32.0)	44.4 (10.3)	NA	21/21 *^c^	21	0.5–3	9
Clément 2021 *^b^ [17]	12–52	NA	NA	POMC/PCSK /LEPR	NA	NA	NA	35/-	35	0.25–3	9
Collet 2017 [12]	4	NA	NA	POMC	110.64 (23.39)	NA	NA	11/5	16	0.01 kg/dia	-
Farooqi 2021 *^d^ [19]	4–12	39.5 (17.6)	NA	POMC/PCSK1/LEPR	NA	50.3 (9.4)	NA	35/-	35	3	8
Farooqi 2022 *^a^ [18]	26–52	NA	NA	POMC/PCSK1/LEPR	142.97 (28.70)	50.2 (9.41)	4.04 (0.65)	35/-	35	NA	8
Gordon 2020 [26]	12	40.5 (8.5)	Male: 22 (26) Fem.: 63 (74)	POMC/LEPR/BBS/ALSTROM	126.6 (20.8)	45.7 (5.6)	NA	61/24	85	2 mg QD 30 mg QW	-
Haqq 2022 [8]	52	19.8 (10.2)	Male: 5 (39) Fem.: 23 (61)	BBS/ ALSTROM	111.7 (30.4)	42.3 (11.0)	NA	19/19	38	3	-
Haws 2020 *^d^ [22]	52	22.5 (14.7)	Male: 4 (40) Fem.: 6 (60)	BBS	128.1 (28.6)	44.8 (4.1)	NA	10/-	10	0.5–3	8
Moreno 2022 *^a^ [20]	26–52	NA	NA	SRC1	139.7 (25.1)	45.4 (11.3)	2.99 (0.63)	30/-	30	NA	8

Int., intervention; Fem., Female; *^a^ The Argente I (2022) [15], Argente II (2022) [16], Moreno (2022) [20], and Farooqi (2022) [18] studies, despite having the same identifier (NCT03651765), have different syndromic populations (SH2B1, BBS, SRC1 and POMC, PSK1, LERP, respectively); *^b^ The Clément (2020) [7] and Clément (2021) [17] studies (NCT02896192 and NCT03287960), despite having overlapping populations, evaluate different outcomes (they evaluate weight and adverse effects, respectively); *^c^ CROSSOVER; *^d^ The Haws (2020) [22] and Farooqi (2021) [19] studies, despite having the same identifier (NCT03013543), have different syndromic populations (BBS and POMC, PCSK1, or LEPR, respectively).

**Table 2:** Statistical analysis of the outcomes of interest.

Outcomes	Studies	No. of Patients	MD or OR	95% CI	Heterogeneity			
					Tau^2^	df	*p*-Value	I^2^ (%)
**Weight loss**	11	197	−6.915	[−8.489, −5.341]	3.939	10	<0.001	92.043
**BMI (** **kg/m^2^)**	7	72	−10.55	[−12.954, −8.161]	4.002	6	0.126	39.756
**BMI** **score (kg/m^2^)**	6	45	−0.610	[−1.372, 0.152]	0.863	5	0.126	99.156
**Adverse events**								
**Hunger score**	5	115	−35.61	[−60.61, −10.61]	779.89	4	<0.001	97.727
**Hyperpigmentation**	6	131	0.692	[0.575, 0.809]	0.013	5	0.008	67.811
**Headache**	5	50	0.277	[0.122, 0.433]	0.025	4	<0.001	81.383
**Nausea**	6	85	0.390	[0.248, 0.533]	0.024	5	<0.001	77.048
**Vomiting**	5	38	0.207	[0.103, 0.312]	0.009	4	0.029	62.957
**ISRs**	4	78	0.816	[0.616, 1.017]	0.028	2	<0.001	88.714
**Sexual dysfunction**	4	12	0.074	[0.014, 0.135]	0.002	3	0.149	43.679

## Data Availability

Not applicable.

## Supplementary Materials

The following supporting information can be downloaded at: https://www.mdpi.com/article/10.3390/jpm13101460/s1. Table S1: Changes in anthropometric, cardiovascular, and metabolic parameters compared with baseline at approximately 1 year while receiving a therapeutic dose of setmelanotide (safety analysis set). Figure S1. Weight loss; Figure S2. Body Mass Index (BMI) score; Figure S3. Hunger Score; Table S2: Safety analysis of the use of Setmelanotide. Figure S4. Headache; Figure S5. Nausea; Figure S6. Vomiting; Figure S7. Sexual dysfunction; Figure S8. Injection site reactions (ISRs); Table S3: Newcastle-ottawa-scale (NOS) assessment; Figure S9. Risk of bias summary for randomized studies (RoB 2). Figure S10. The level of evidence for weight loss was classified according to the GRADE tool. Figure S11. Weight loss leave one out analysis; Figure S12. BMI leave one out analysis; Figure S13. BMI score leave one out analysis; Figure S14. ISRs leave one out analysis; Figure S15. Skin hyperpigmentation leave one out analysis.

## Abbreviations

Adverse effects: AEs; Bardet–Biedl syndrome: BBS; Body mass index: BMI; Grading of Recommendations, Assessment, Development, and Evaluation: GRADE; Food and Drug Administration: FDA; Injection site reactions: ISRs; Leptin receptor deficiency: LEPR; Mean difference: MD; Melanocortin-4 receptor: MC4R; Newcastle–Ottawa Scale: NOS; Odds ratio: OR; Preferred Reporting Items for Systematic Reviews and Meta-Analysis: PRISMA; Proopiomelanocortin: POMC; Prospective International Registry of Systematic Reviews: PROSPERO; Protein convertase subtilisin/kexin type 1: PCSK1; Randomized clinical trials: RCTs; World Health Organization: WHO.
