# Supplementary material for: Efficacy and Safety of Setmelanotide, a Melanocortin-4 Receptor Agonist, for Obese Patients: A Systematic Review and Meta-Analysis
Source: J Pers Med. 2023 Oct 4;13(10):1460. doi: 10.3390/jpm13101460 (PMC10608339; doi:10.3390/jpm13101460)
Supplement: Supplementary file 1 [file jpm-13-01460-s001.zip › jpm-2618282-supplementary.pdf]

Supplementary Data

Table S1: Changes in anthropometric, cardiovascular, and metabolic parameters compared with baseline at approximately 1 year while receiving a therapeutic dose of setmelanotide (safety analysis set).

	ACTIVE TREATMENT BASELINE	PERCENTAGE CHANGE FROM BASELINE (END OF TREATMENT)	N
**Anthropometric and clinical parameters**			
Hunger score †	5.93 (2.63)	-28.0 % (30.86)	115
Waist circumference, cm*a	119.75 (18.52)	-6.311 % (8.50)	63
**Lipids, mg/dl*c**			
Hdl cholesterol	17.80 (22.19)	14.0852% (26.34)	61
Ldl cholesterol	43.44 (50.47)	-8.0016% (23.65)	62
Triglycerides	17.80 (22.19)	-16.3839% (30.93)	62
**Cardiovascular parameters, mm hg*d**			
Diastolic blood pressure	71.93 (10.68)	-2.67% (13.39)	26
Systolic blood pressure	114.53 (11.04)	0.54 % (10.59)	26

† 11-point Likert-type scale - population denominator (n=115) ([7,16,18,21,24] studies); *a, population denominator (n=63) ([7,11,16] studies); *c, population denominator (n=61) ([7,16,21] studies); *d, population denominator (n=26) ([16,21] studies);

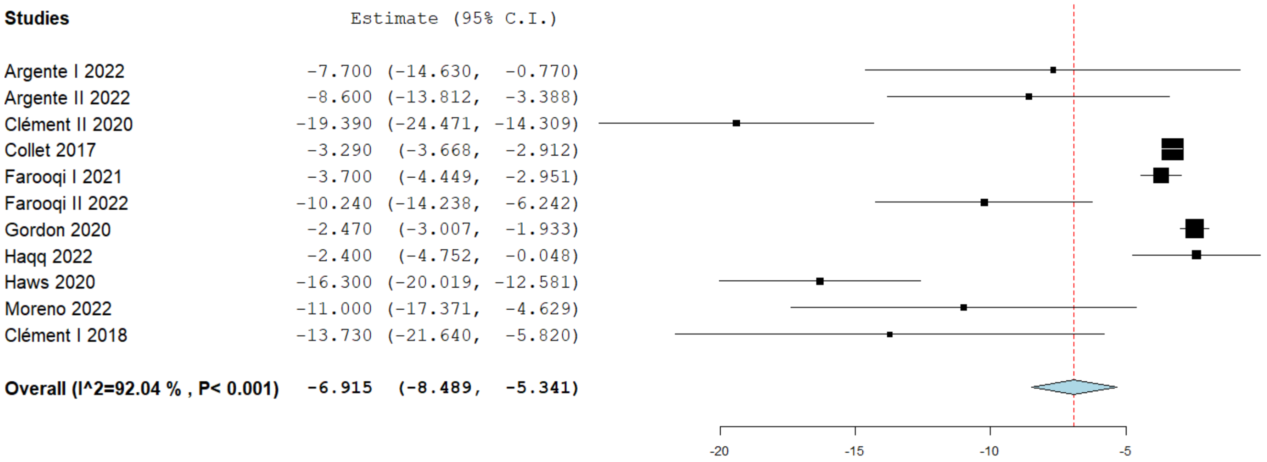

Figure S1. Weight loss; (OR -6.91; 95% CI -8.48, -5.34; p<0.001).

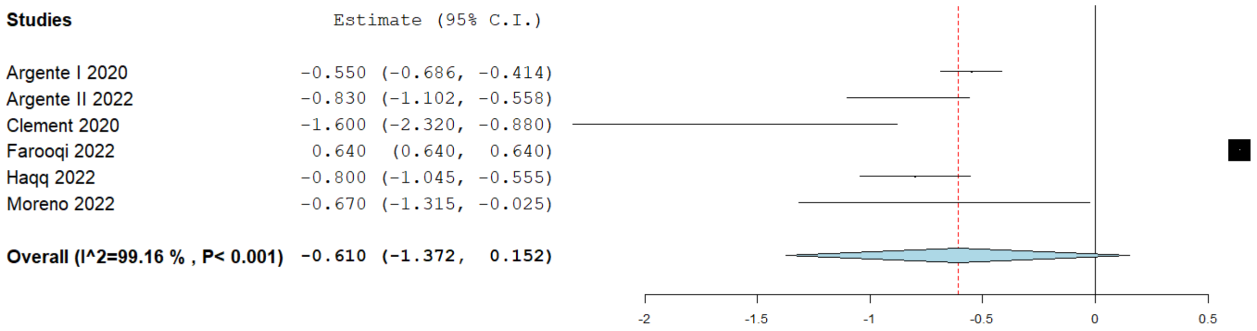

Figure S2. Body Mass Index (BMI) score; (OR -0.61; 95% CI -1.37, 0.15; p<0.001).

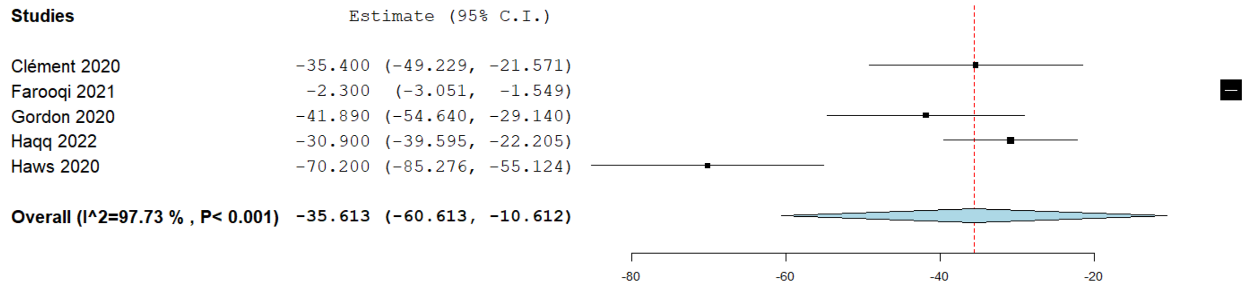

Figure S3. Hunger Score; (OR -35.6; 95% CI -60.6, -10.6; p<0.001).

Table S2 : Safety analysis of the use of Setmelanotide.

ADVERSE EVENTS	INTERVENTION (N190)
Skin hyperpigmentation	131 (68,9%)
Nausea	85(44,7%)
ISRs	78(41,05%)
Headache	50(26,3%)
Injection site erythema	42(22,1%)
Vomiting	38(20%)
Injection site pruritus	26(13,6%)
Injection site pain	12(6,3%)
Injection site bruising	11(5,7%)
Sexual disorders [*a]	12(14,28%%)
Injection site induration	9(4,7%)
Diarrhoea	8(4,2%)
Back pain	4(2,1%)
Cough	4(2,1%)
Hdl cholesterol decrease	4(2,1%)
Injection site edema	4(2,1%)
Melanocytic nevus	4(2,1%)
Nasopharyngitis	4(2,1%)
Abdominal pain	4(2,1%)
	N179
Treatment-related adverse events	177 (98,8%)
Serious adverse events [*b]	22 (12,2%)
Serious treatment-related adverse events	1 (0,5%)
Adverse events leading to study drug withdrawal [*c]	14 (5,22%)
Adverse events leading to death	0 (0%)

[*a] Denominator of the male population (n=84) ([7,11,16] studies). [*b] One patient had a myocardial infarction and gastrointestinal haemorrhage which were considered unrelated to setmelanotide.[*c] Total denominator of 268 patients; one patient with BBS had an anaphylactic reaction to the study drug (placebo). The other adverse effects were also not considered to be related to treatment with Setmelanotide.

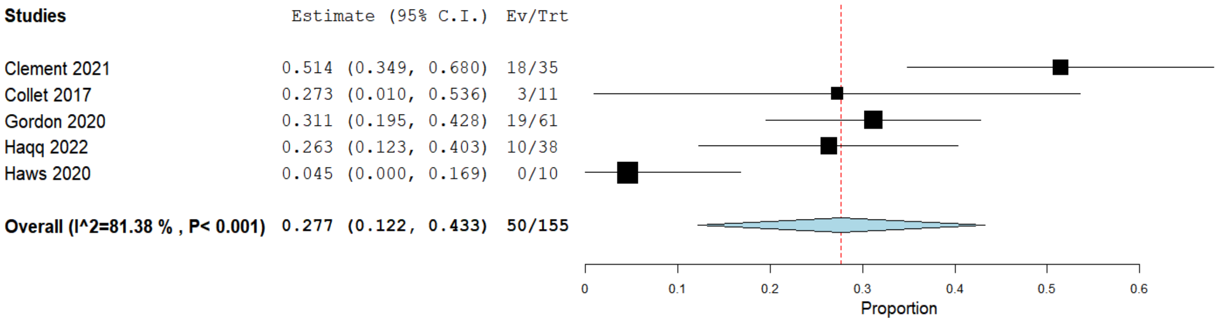

Figure S4. Headache; (OR 0.27; 95% CI 0.12, 0.43; p<0.001).

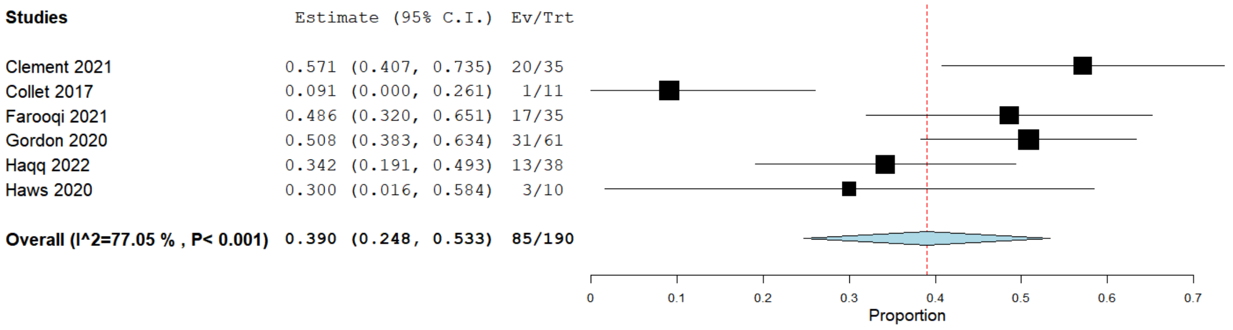

Figure S5. Nausea; (OR 0.39; 95% CI 0.24, 0.53; p<0.001).

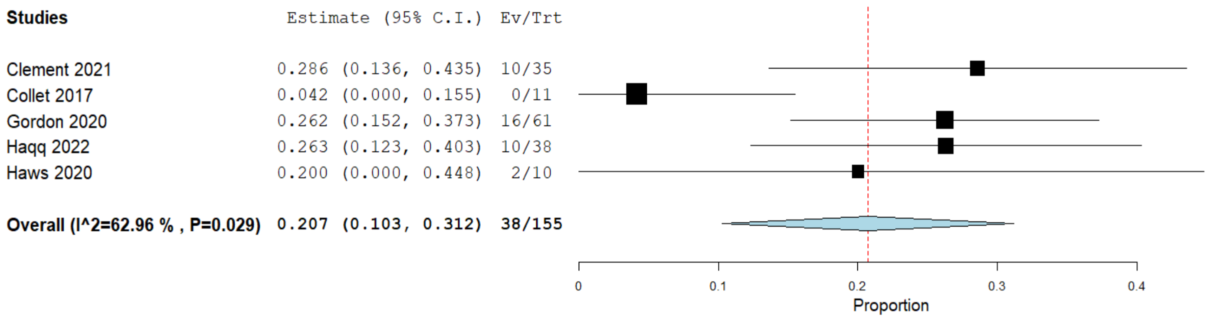

Figure S6. Vomiting; (OR 0.20; 95% CI 0.10, 0.31; p=0.029).

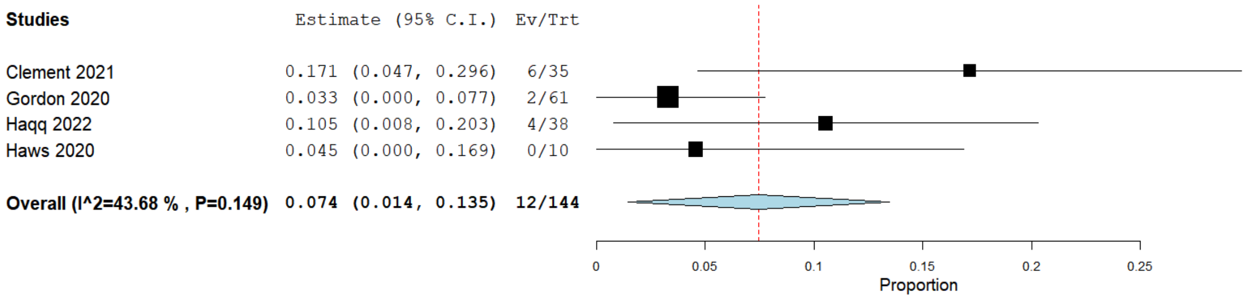

Figure S7. Sexual dysfunction; (OR -0.074; 95% CI 0.01, 0.13; p=0.149).

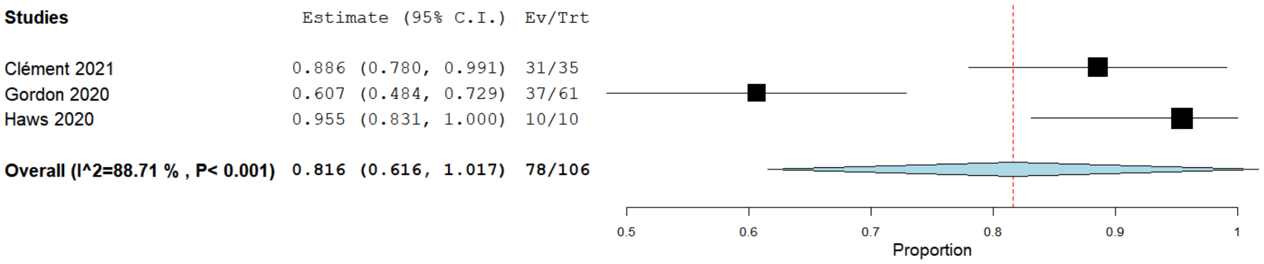

Figure S8. Injection site reactions (ISRs) ; (OR 0.81; 95% CI 0.61, 1.01; p<0.001).

Table S3: Newcastle-ottawa-scale (NOS) assessment;

NOS FOR THE RISK OF BIAS AND QUALITY ASSESSMENT OF NRSS

**AUTHOR**	YEAR	SELECTION				COMPARABILITY	OUCTOME			TOTAL SCORE
		1	2	3	4	1	1	2	3	
Argent I* [14]	2022	★	-	★	★	★★	★	★	★	8
Argent II* [15]	2022	★	-	★	★	★★	★	★	★	8
Clément [20]	2018	★	-	★	★	★★	★	★	★	8
Clément** [6]	2020	★	★	★	★	★★	★	★	★	9
Clément** [16]	2021	★	★	★	★	★★	★	★	★	9
Farooqi*** [18]	2021	★	-	★	★	★★	★	★	★	8
Farooqi* [17]	2022	★	-	★	★	★★	★	★	★	8
Haws*** [21]	2020	★	-	★	★	★★	★	★	★	8
Moreno* [19]	2022	★	-	★	★	★★	★	★	★	8

* The studies Argente I (2022) [14], Argente II (2022) [15], Moreno (2022) [19], and Farooqi (2022) [17], despite sharing the same National Clinical Trial (NCT) identifier, encompass distinct syndromic populations (SH2B1, BBS, SRC1, and POMC, PSK1, LERP, respectively). **The studies Clément (2020) [6], and Clément (2021) [16], despite having an overlapping population, assess different outcomes (weight and adverse effects, respectively). *** The studies Haws (2020) [21], and Farooqi (2021) [18], although sharing the same National Clinical Trial (NCT) identifier, involve distinct syndromic populations (BBS and POMC, PCSK1, or LEPR-related, respectively)./One of the criteria for inclusion of patients for long-term follow-up in the Farroqi [17,18], and Argente [14,15] clinical trials was that they had previously participated in an evaluation of the efficacy and safety of setmelanotide and had achieved positive results in terms of weight loss. According to the first observation, these three studies are part of the same clinical trial, but each study evaluates patients with different genetic syndromes.

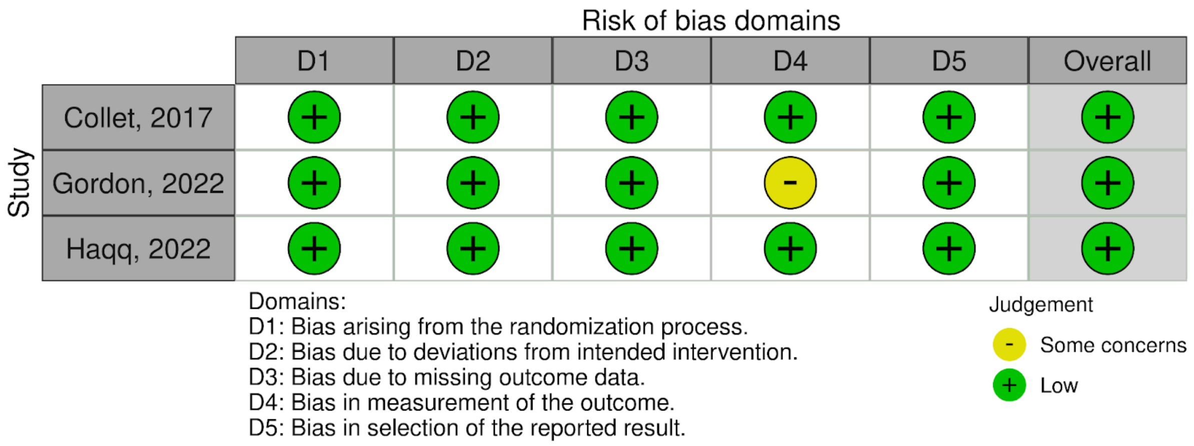

Figure S9 - Risk of bias summary for randomized studies (RoB 2).

Certainty assessment							№ of patients		Effect		Certainty	Importance
**№ of studies**	**Study design**	**Risk of bias**	**Inconsistency**	**Indirectness**	**Imprecision**	**Other considerations**	**Setmelanotide**	**placebo**	**Relative (95% CI)**	**Absolute (95% CI)**		
**Novo desfecho (follow-up: range 4 weeks to 14 weeks)**												
4	randomised trials	not serious	serious[a]	not serious	not serious	strong association	109	67	-	mean **3.52 fewer** (3.98 fewer to 3.05 fewer)	⊕⊕⊕⊕ High	IMPORTANTE

CI: confidence interval

Explanations

a. This result showed a high level of heterogeneity, partly explained by the different populations included, the dose/time of treatment and the inclusion of an observational study (as recommended by Cochrane, the level of evidence in good quality studies should start high, as future analyses will already have an adequate level of downgrading).

Figure S10. The level of evidence for weight loss was classified according to the GRADE tool.

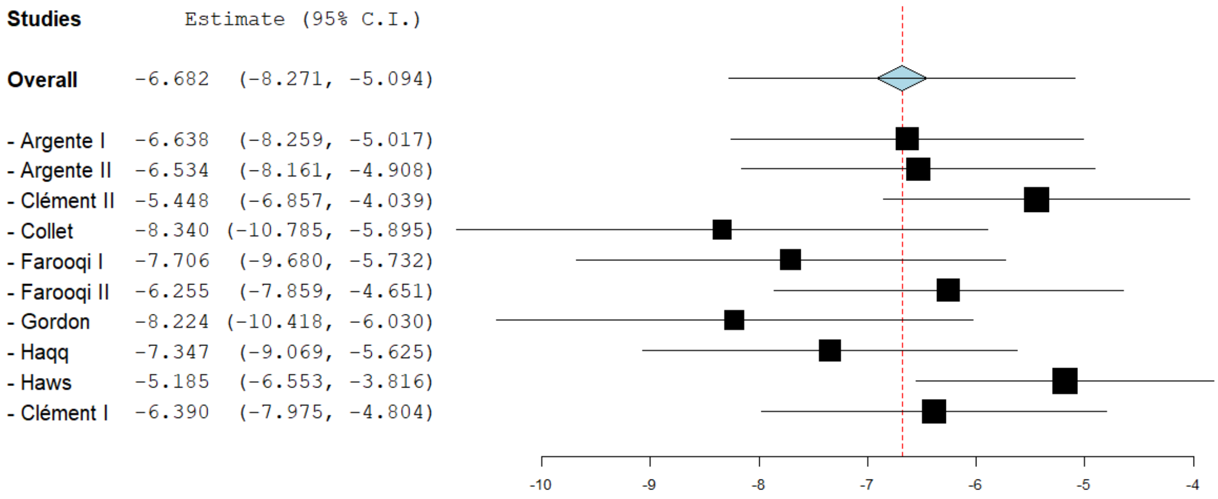

Figure S11. Weight loss leave one out analysis; (OR -6.68; 95% CI -8.27, -5.09).

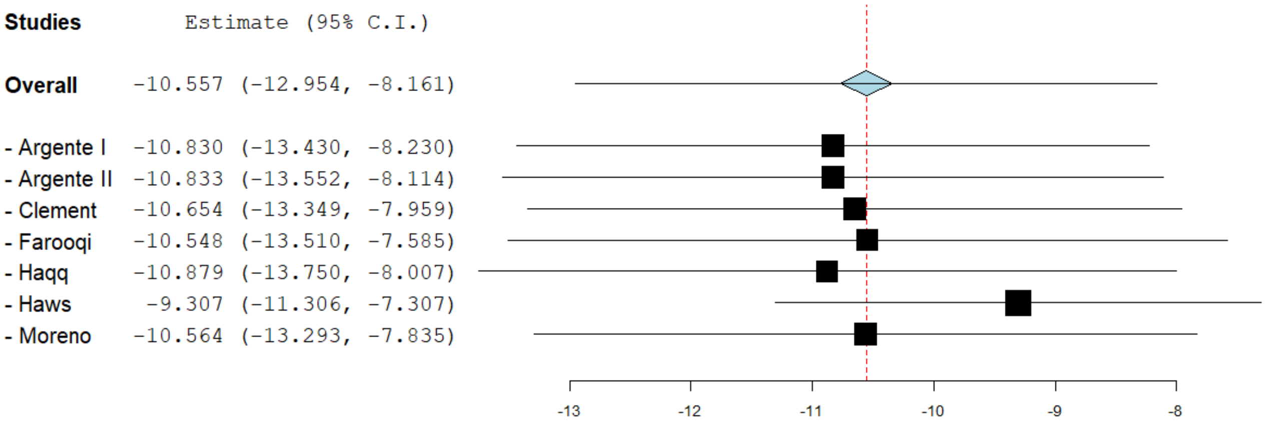

Figure S12. BMI leave one out analysis; (OR -10.55; 95% CI -12.95, -8.16).

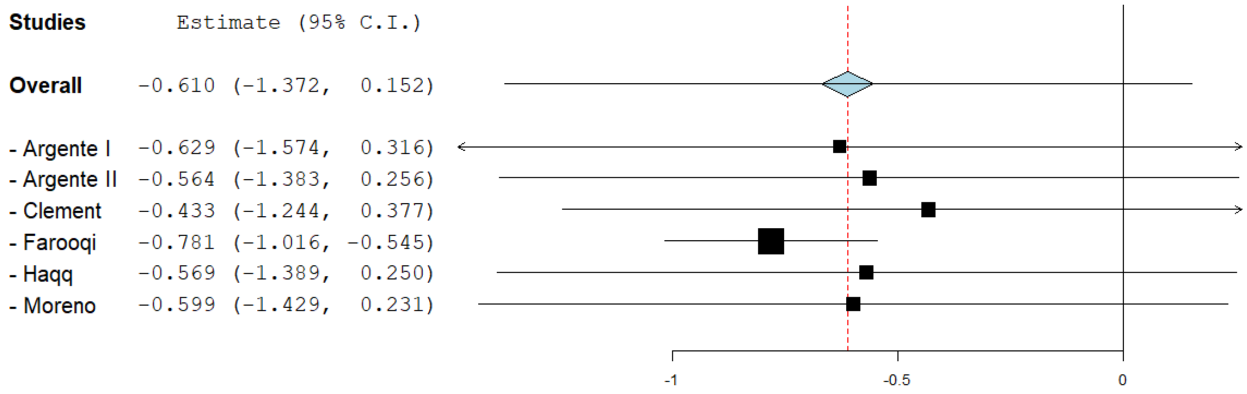

Figure S13. BMI score leave one out analysis; (OR -0.61; 95% CI -1.37, 0.15).

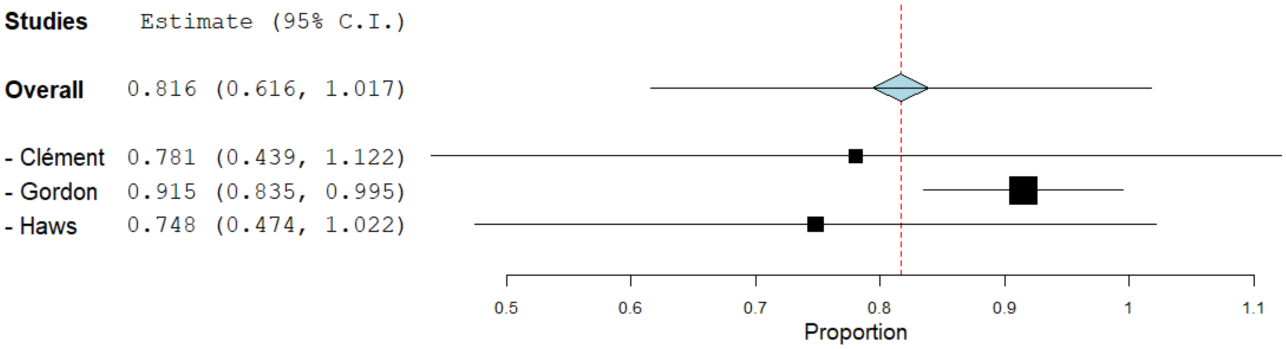

Figure S14. ISRs leave one out analysis; (OR 0.81; 95% CI 0.61, 1.01).

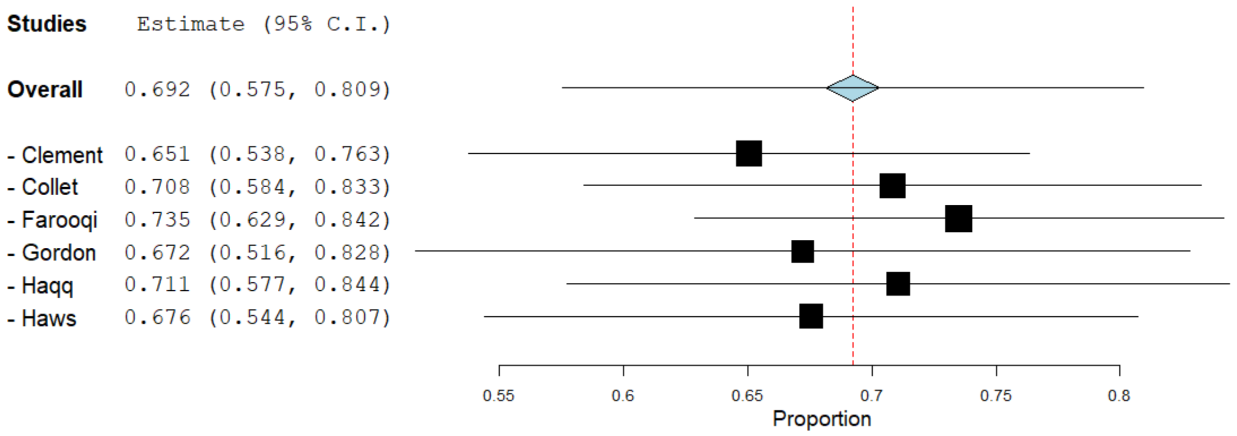

Figure S15. Skin hyperpigmentation leave one out analysis; (OR 0.69; 95% CI 0.57, 0.80).

References

1. Mayoral, L.-C.; Andrade, G.; Mayoral, E.-C.; Huerta, T.; Canseco, S.; Rodal Canales, F.; Cabrera-Fuentes, H.; Cruz, M.; Pérez Santiago, A.; Alpuche, J.; et al. Obesity Subtypes, Related Biomarkers & Heterogeneity. *Indian J. Med. Res.* **2020**, *151*, 11. https://doi.org/10.4103/ijmr.IJMR_1768_17.
2. Dia Mundial da Obesidade 2022: Acelerar Ação Para Acabar Com a Obesidade—OPAS/OMS | Organização Pan-Americana da Saúde. Available online: https://www.paho.org/pt/noticias/4-3-2022-dia-mundial-da-obesidade-2022-acelerar-acao-para-acabar-com-obesidade (accessed on 22 March 2023).
3. World Obesity Day Atlases | Obesity Atlas 2023. Available online: https://data.worldobesity.org/publications/?cat=19 (accessed on 9 September 2023).
4. Melchior, V.; Fuchs, S.; Scantamburlo, G. Obesity and eating disorders. *Rev. Med. Liege* **2021**, *76*, 134–139.
5. De Lorenzo, A.; Romano, L.; Di Renzo, L.; Di Lorenzo, N.; Cenname, G.; Gualtieri, P. Obesity: A Preventable, Treatable, but Relapsing Disease. *Nutr. Burbank Los Angel. Cty. Calif* **2020**, *71*, 110615. https://doi.org/10.1016/j.nut.2019.110615.
6. Cignarella, A.; Busetto, L.; Vettor, R. Pharmacotherapy of Obesity: An Update. *Pharmacol. Res.* **2021**, *169*, 105649. https://doi.org/10.1016/j.phrs.2021.105649.
7. Clément, K.; van den Akker, E.; Argente, J.; Bahm, A.; Chung, W.K.; Connors, H.; De Waele, K.; Farooqi, I.S.; Gonneau-Lejeune, J.; Gordon, G.; et al. Efficacy and Safety of Setmelanotide, an MC4R Agonist, in Individuals with Severe Obesity Due to LEPR or POMC Deficiency: Single-Arm, Open-Label, Multicentre, Phase 3 Trials. *Lancet Diabetes Endocrinol.* **2020**, *8*, 960–970. https://doi.org/10.1016/S2213-8587(20)30364-8.
8. Haqq, A.M.; Chung, W.K.; Dollfus, H.; Haws, R.M.; Martos-Moreno, G.Á.; Poitou, C.; Yanovski, J.A.; Mittleman, R.S.; Yuan, G.; Forsythe, E.; et al. Efficacy and Safety of Setmelanotide, a Melanocortin-4 Receptor Agonist, in Patients with Bardet-Biedl Syndrome and Alström Syndrome: A Multicentre, Randomised, Double-Blind, Placebo-Controlled, Phase 3 Trial with an Open-Label Period. *Lancet Diabetes Endocrinol.* **2022**, *10*, 859–868. https://doi.org/10.1016/S2213-8587(22)00277-7.
9. Ju, S.H.; Yun, H.; Oh, Y.; Choi, Y.; Sohn, J.-W. Melanocortin-4 Receptors Activate Sympathetic Preganglionic Neurons and Elevate Blood Pressure via TRPV1. *Cell Rep.* **2022**, *41*, 111579. https://doi.org/10.1016/j.celrep.2022.111579.
10. Stutzmann, F.; Tan, K.; Vatin, V.; Dina, C.; Jouret, B.; Tichet, J.; Balkau, B.; Potoczna, N.; Horber, F.; O'Rahilly, S.; et al. Prevalence of Melanocortin-4 Receptor Deficiency in Europeans and Their Age-Dependent Penetrance in Multigenerational Pedigrees. *Diabetes* **2008**, *57*, 2511–2518. https://doi.org/10.2337/db08-0153.
11. Farooqi, I.S.; Keogh, J.M.; Yeo, G.S.H.; Lank, E.J.; Cheetham, T.; O'Rahilly, S. Clinical Spectrum of Obesity and Mutations in the Melanocortin 4 Receptor Gene. *N. Engl. J. Med.* **2003**, *348*, 1085–1095. https://doi.org/10.1056/NEJMoa022050.
12. Collet, T.-H.; Dubern, B.; Mokrosinski, J.; Connors, H.; Keogh, J.M.; Mendes de Oliveira, E.; Henning, E.; Poitou-Bernert, C.; Oppert, J.-M.; Tounian, P.; et al. Evaluation of a Melanocortin-4 Receptor (MC4R) Agonist (Setmelanotide) in MC4R Deficiency. *Mol. Metab.* **2017**, *6*, 1321–1329. https://doi.org/10.1016/j.molmet.2017.06.015.
13. Pressley, H.; Cornelio, C.K.; Adams, E.N. Setmelanotide: A Novel Targeted Treatment for Monogenic Obesity. *J. Pharm. Technol. JPT Off. Publ. Assoc. Pharm. Tech.* **2022**, *38*, 368–373. https://doi.org/10.1177/87551225221116010.
14. Ottawa Hospital Research Institute. Available online: https://www.ohri.ca/programs/clinical_epidemiology/oxford.asp (accessed on 10 August 2023).
15. Argente, J.; Beales, P.; Clément, K.; Dollfus, H.; Forsythe, E.; Haqq, A.; Haws, R.; Martos-Moreno, G.; Mittleman, R.; Yanovski, J.; et al. ODP606 Long-Term Efficacy of Setmelanotide in Patients With Bardet-Biedl Syndrome. *J. Endocr. Soc.* **2022**, *6*, A14. https://doi.org/10.1210/jendso/bvac150.029.
16. Argente, J.; Farooqi, S.; Chung, W.; Wabitsch, M.; Scimia, C.; Srinivasan, M.; Hu, S. RF24 | PSUN91 Body Mass Index and Weight Reduction in Patients With SH2B1 Genetic Variant Obesity After One Year of Setmelanotide. *J. Endocr. Soc.* **2022**, *6*, A35–A35. https://doi.org/10.1210/jendso/bvac150.073.
17. Karine Clément, MD, PhD, Erica L T van den Akker, MD, PhD, Gregory Gordon, MD, Guojun Yuan, PhD, Peter Kühnen, MD Timing of Onset of Adverse Events With Setmelanotide, an MC4R Agonist, in Patients With Severe Obesity Due to LEPR or POMC Deficiency | Journal of the Endocrine Society | Oxford Academic. Available online: https://academic.oup.com/jes/article/5/Supplement_1/A30/6240595 (accessed on 27 August 2023).
18. Farooqi, S.; Miller, J.; Ohayan, O.; Scimia, C.; Still, C.; Yohn, M.; Yuan, G.; Argente, J.; Buckley, B. OR10-1 Body Mass Index and Weight Reductions in Patients With Obesity Due to Heterozygous Variants in POMC, PCSK1, and LEPR After 1 Year of Setmelanotide. *J. Endocr. Soc.* **2022**, *6*, A15. https://doi.org/10.1210/jendso/bvac150.031.
19. Farooqi, S.; Miller, J.L.; Ohayon, O.; Yuan, G.; Stewart, M.; Scimia, C.; Yanovski, J. Effects of Setmelanotide in Patients With POMC, PCSK1, or LEPR Heterozygous Deficiency Obesity in a Phase 2 Study. *J. Endocr. Soc.* **2021**, *5*, A669–A670. https://doi.org/10.1210/jendso/bvab048.1367.
20. Martos-Moreno, G.Á.; Argente, J.; Scimia, C.; Ohayan, O.; Yuan, G.; Farooqi, S. ODP605 Body Mass Index and Weight Reductions in Patients With SRC1 Genetic Variant Obesity After 1 Year of Setmelanotide. *J. Endocr. Soc.* **2022**, *6*, A13–A14. https://doi.org/10.1210/jendso/bvac150.028.
21. Clément, K.; Biebermann, H.; Farooqi, I.S.; Van Der Ploeg, L.; Wolters, B.; Poitou, C.; Puder, L.; Fiedorek, F.; Gottesdiener, K.; Kleinau, G.; et al. MC4R Agonism Promotes Durable Weight Loss in Patients with Leptin Receptor Deficiency. *Nat. Med.* **2018**, *24*, 551–555. https://doi.org/10.1038/s41591-018-0015-9.

22. Haws, R.; Brady, S.; Davis, E.; Fletty, K.; Yuan, G.; Gordon, G.; Stewart, M.; Yanovski, J. Effect of Setmelanotide, a Melanocortin-4 Receptor Agonist, on Obesity in Bardet-Biedl Syndrome. *Diabetes Obes. Metab.* **2020**, *22*, 2133–2140. https://doi.org/10.1111/dom.14133.
23. RoB 2: A Revised Cochrane Risk-of-Bias Tool for Randomized Trials|Cochrane Bias. Available online: https://methods.cochrane.org/bias/resources/rob-2-revised-cochrane-risk-bias-tool-randomized-trials (accessed on 27 January 2023).
24. GRADEpro. Available online: https://www.gradepro.org/ (accessed on 4 May 2023).
25. OpenMeta[Analyst]—CEBM @ Brown. Available online: http://www.cebm.brown.edu/openmeta/ (accessed on 10 August 2023).
26. Gordon, G.; Valles-Sukkar, A.; Yuan, G.; Stewart, M. A Randomized Trial of a Once-Weekly Formulation of Setmelanotide in Individuals with Obesity|Cochrane Library. Available online: https://www.cochranelibrary.com/central/doi/10.1002/central/CN-02259856/full (accessed on 5 May 2023).
27. Kanti, V.; Puder, L.; Jahnke, I.; Krabusch, P.M.; Kottner, J.; Vogt, A.; Richter, C.; Andruck, A.; Lechner, L.; Poitou, C.; et al. A Melanocortin-4 Receptor Agonist Induces Skin and Hair Pigmentation in Patients with Monogenic Mutations in the Leptin-Melanocortin Pathway. *Skin Pharmacol. Physiol.* **2021**, *34*, 307–316. https://doi.org/10.1159/000516282.
28. Angelidi, A.M.; Belanger, M.J.; Kokkinos, A.; Koliaki, C.C.; Mantzoros, C.S. Novel Noninvasive Approaches to the Treatment of Obesity: From Pharmacotherapy to Gene Therapy. *Endocr. Rev.* **2022**, *43*, 507–557. https://doi.org/10.1210/endrev/bnab034.
29. Loos, R.J.F.; Janssens, A.C.J.W. Predicting Polygenic Obesity Using Genetic Information. *Cell Metab.* **2017**, *25*, 535–543. https://doi.org/10.1016/j.cmet.2017.02.013.
30. Variability in the Heritability of Body Mass Index: A Systematic Review and Meta-Regression—PMC. Available online: https://www.ncbi.nlm.nih.gov/pmc/articles/PMC3355836/ (accessed on 27 August 2023).
31. Kühnen, P.; Clément, K.; Wiegand, S.; Blankenstein, O.; Gottesdiener, K.; Martini, L.L.; Mai, K.; Blume-Peytavi, U.; Grüters, A.; Krude, H. Proopiomelanocortin Deficiency Treated with a Melanocortin-4 Receptor Agonist. *N. Engl. J. Med.* **2016**, *375*, 240–246. https://doi.org/10.1056/NEJMoa1512693.
32. Ryan, D.H. Drugs for Treating Obesity. *Handb. Exp. Pharmacol.* **2022**, *274*, 387–414. https://doi.org/10.1007/164_2021_560.
33. Yanovski, S.Z.; Yanovski, J.A. Progress in Pharmacotherapy for Obesity. *JAMA* **2021**, *326*, 129–130. https://doi.org/10.1001/jama.2021.9486.
34. Khera, R.; Murad, M.H.; Chandar, A.K.; Dulai, P.S.; Wang, Z.; Prokop, L.J.; Loomba, R.; Camilleri, M.; Singh, S. Association of Pharmacological Treatments for Obesity With Weight Loss and Adverse Events. *JAMA* **2016**, *315*, 2424–2434. https://doi.org/10.1001/jama.2016.7602.
35. Smits, M.M.; Van Raalte, D.H. Safety of Semaglutide. *Front. Endocrinol.* **2021**, *12*, 645563. https://doi.org/10.3389/fendo.2021.645563.
36. Wilding, J.P.H.; Batterham, R.L.; Calanna, S.; Davies, M.; Van Gaal, L.F.; Lingvay, I.; McGowan, B.M.; Rosenstock, J.; Tran, M.T.D.; Wadden, T.A.; et al. Once-Weekly Semaglutide in Adults with Overweight or Obesity. *N. Engl. J. Med.* **2021**, *384*, 989–1002. https://doi.org/10.1056/NEJMoa2032183.
37. Ladenheim, E.E. Liraglutide and Obesity: A Review of the Data so Far. *Drug Des. Devel. Ther.* **2015**, *9*, 1867–1875. https://doi.org/10.2147/DDDT.S58459.
38. Lin, C.-H.; Shao, L.; Zhang, Y.-M.; Tu, Y.-J.; Zhang, Y.; Tomlinson, B.; Chan, P.; Liu, Z. An Evaluation of Liraglutide Including Its Efficacy and Safety for the Treatment of Obesity. *Expert Opin. Pharmacother.* **2020**, *21*, 275–285. https://doi.org/10.1080/14656566.2019.1695779.
39. O'Neil, P.M.; Birkenfeld, A.L.; McGowan, B.; Mosenzon, O.; Pedersen, S.D.; Wharton, S.; Carson, C.G.; Jepsen, C.H.; Kabisch, M.; Wilding, J.P.H. Efficacy and Safety of Semaglutide Compared with Liraglutide and Placebo for Weight Loss in Patients with Obesity: A Randomised, Double-Blind, Placebo and Active Controlled, Dose-Ranging, Phase 2 Trial. *Lancet* **2018**, *392*, 637–649. https://doi.org/10.1016/S0140-6736(18)31773-2.
40. Rubino, D.M.; Greenway, F.L.; Khalid, U.; O'Neil, P.M.; Rosenstock, J.; Sørrig, R.; Wadden, T.A.; Wizert, A.; Garvey, W.T.; STEP 8 Investigators; et al. Effect of Weekly Subcutaneous Semaglutide vs Daily Liraglutide on Body Weight in Adults With Overweight or Obesity Without Diabetes: The STEP 8 Randomized Clinical Trial. *JAMA* **2022**, *327*, 138. https://doi.org/10.1001/jama.2021.23619.
41. Forsythe, E.; Haws, R.M.; Argente, J.; Beales, P.; Martos-Moreno, G.Á.; Dollfus, H.; Chirila, C.; Gnanasakthy, A.; Buckley, B.C.; Mallya, U.G.; et al. Quality of Life Improvements Following One Year of Setmelanotide in Children and Adult Patients with Bardet–Biedl Syndrome: Phase 3 Trial Results. *Orphanet J. Rare Dis.* **2023**, *18*, 12. https://doi.org/10.1186/s13023-022-02602-4.
42. Rhythm Pharmaceuticals Optimizes Design of EMANATE and DAYBREAK Clinical Trials to Advance Setmelanotide for Rare Genetic Diseases of Obesity|Rhythm Pharmaceuticals, Inc. Available online: https://ir.rhythmtx.com/news-releases/news-release-details/rhythm-pharmaceuticals-optimizes-design-emanate-and-daybreak/ (accessed on 20 August 2023).
